# Flowering and fruit-set in cassava under extended red-light photoperiod supplemented with plant-growth regulators and pruning

**DOI:** 10.1186/s12870-023-04349-x

**Published:** 2023-06-23

**Authors:** Julius K. Baguma, Settumba B Mukasa, Ephraim Nuwamanya, Titus Alicai, Christopher Omongo, Peter T. Hyde, Tim L. Setter, Mildred Ochwo-Ssemakula, William Esuma, Michael Kanaabi, Paula Iragaba, Yona Baguma, Robert S. Kawuki

**Affiliations:** 1 School of Agricultural Sciences, Makerere University, P. O. Box 7062, Kampala, Uganda; 2 National Crops Resources Research Institute (NaCRRI), Namulonge, P. O. Box 7084, Kampala, Uganda; 3 Soil and Crop Sciences, School of Integrative Plant Science, Cornell University, Ithaca, NY USA; 4 National Agricultural Research Organisation (NARO) Secretariat, P. O. Box 295, Entebbe, Uganda

**Keywords:** Cassava breeding, Plant growth regulators, Floral induction, Fruit-set, Photoperiod

## Abstract

**Background:**

Cassava (*Manihot esculenta* Crantz) is staple food and major source of calories for over 500 million people in sub-Saharan Africa. The crop is also a source of income for smallholder farmers, and has increasing potential for industrial utilization. However, breeding efforts to match the increasing demand of cassava are impeded by its inability to flower, delayed or unsynchronized flowering, low proportion of female flowers and high fruit abortions. To overcome these sexual reproductive bottlenecks, this study investigated the effectiveness of using red lights to extend the photoperiod (RLE), as a gateway to enhancing flowering and fruit set under field conditions.

**Materials and methods:**

Panels of cassava genotypes, with non- or late and early flowering response, 10 in each case, were subjected to RLE from dusk to dawn. RLE was further evaluated at low (LL), medium (ML) and high (HL) red light intensities, at ~  ≤ 0.5; 1.0 and 1.5PFD (Photon Flux Density) in µmol m^−2^ s^−1^ respectively. Additionally, the effect of a cytokinin and anti-ethylene as plant growth regulators (PGR) and pruning under RLE treatment were examined.

**Results:**

RLE stimulated earlier flower initiation in all genotypes, by up to 2 months in the late-flowering genotypes. Height and number of nodes at first branching, particularly in the late-flowering genotypes were also reduced, by over 50%. Number and proportion of pistillate flowers more than doubled, while number of fruits and seeds also increased. Number of branching levels during the crop season also increased by about three. Earlier flowering in many genotypes was most elicited at LL to ML intensities. Additive effects on flower numbers were detected between RLE, PGR and pruning applications. PGR and pruning treatments further increased number and proportion of pistillate flowers and fruits. Plants subjected to PGR and pruning, developed bisexual flowers and exhibited feminization of staminate flowers. Pruning at first branching resulted in higher pistillate flower induction than at second branching.

**Conclusions:**

These results indicate that RLE improves flowering in cassava, and its effectiveness is enhanced when PGR and pruning are applied. Thus, deployment of these technologies in breeding programs could significantly enhance cassava hybridizations and thus cassava breeding efficiency and impact.

**Supplementary Information:**

The online version contains supplementary material available at 10.1186/s12870-023-04349-x.

## Introduction

Cassava (*Manihot esculenta* Crantz) is a staple crop and one of the main sources of calories, contributing to the nutrition and livelihood of over 500 million people in sub-Saharan Africa [1–3]. It is also a food security crop and potentially important in spurring industrial development as well as raising incomes for the rural poor farmers, processors and traders, particularly in developing countries [1–5]. Furthermore, it is a crop resilient to unpredictable climate variabilities and their extremes as well as poor soils [2, 6]. Thus, its continued genetic improvement targeting both food and industrial use as well as tolerance to the current threats of climate change is crucial. Fortunately, there are current breeding efforts aimed at improving the crop using modern tools such as genomic selection [7]. These efforts aim at development and deployment of cassava varieties that meet quality requirements of smallholder farmers [8]. Attainment of this goal requires timely crossing or hybridization of elite progenitors to obtain recombinant and/or segregant seed with the desired trait combinations followed by selection of high performance genotypes.

However, successful crossing or hybridization in cassava breeding is impeded by the inability of a high fraction of genotypes to flower or produce sufficient flowers. Additionally, cassava is commonly propagated vegetatively, thus phenotypic selections in the crop are not usually based on flowering and seed traits. Truly, some cassava genotypes flower profusely, but others are poor at flowering while others never flower at all [9, 10] and thus complicating crossing programs. For the flowering genotypes, time to flowering and fruiting varies markedly [10, 11] making synchronization of flowering and crossing difficult [10, 12]. Additionally, the ratio of female to male flowers per inflorescence is small [10, 13, 14]. Success of controlled pollination is usually low, one or two seeds per flower [9, 15]. Unsynchronized, poor or delayed flowering coupled with high flower and fruit abortion rates (before production of viable seed) largely underpin impediments of cassava hybridization [9, 15, 16].

The majority of farmers prefer erect and late-branching cassava genotypes due to their compatibility to some agronomic practices, e.g. intercropping, weed control as well as ability to obtain sufficient planting materials. Unfortunately these genotypes have no or low flowering capacity, thus limiting their usage as progenitors. These flowering impediments necessitate development of an efficient protocol to induce or enhance flowering so as to allow breeders to fully utilize the cassava genetic resources to meet priority food and non-food uses.

Transition of the apical meristem into a floral bud and thus flower production has been artificially induced in a wide range of plants by a number of techniques. Notable of these include application of plant growth regulators (PGRs) [17, 18]; inoculation of a flowering promoter sequence via a viral vector [19, 20]; over-expression of *FLOWERING LOCUS T* (*atFT*) [21, 22]; photoperiod extension [23]; grafting [24] and pruning [25]. These techniques have, to various depths, been explored for flower induction in cassava. For example, application of PGRs such as silver thiosulphate (STS) [26]; over-expression of the *atFT* gene [27–29]; pruning young branches [30, 31]; grafting [12, 32] as well as extension of photoperiod [33, 34].

Flowering enhancement such as through extension of photoperiod is one of the speed breeding techniques [35, 36]. Speed breeding aims to shorten crop breeding cycles through increasing generation cycles so as to hasten crop improvement and food production to meet the growing food demand escalated by the rapidly growing human population [36–38]. Cassava, a critical crop for overcoming food insecurity ought to benefit from these speed breeding innovations.

Speed breeding using photoperiod extension was reported in chickpea (*Cicer arietinum* L*.*) [39]*.* In this crop, photoperiod extension throughout the night (18:00 to 6:00.) caused a reduction in flowering time. Manipulation of photoperiod and light quality or its spectral distribution are now being used to artificially elicit flowering in many crop plants [40, 41]. Extension of photoperiod, by lighting at the end of the natural photoperiod or interrupting dark period with night breaks creates artificial long days which promote flowering in long day plants [40]. The flowering is promoted most when the lighting contains red (R) light with a lower proportion of far red (FR) light than when FR is lacking [40]. For example, extended day length exposure or night break treatments caused earlier flowering in *Arabidopsis* [23] but delayed flower initiation in tomato plants [42] and *Chrysanthemum morifolium* cv. “Radost” [43]. In cassava, early efforts towards photoperiodic induction of flowering were demonstrated by [44]. His observations showed shortened flowering and forking time under natural conditions of longer photoperiods, and the recent reports [33, 34] corroborate this observation. However, the effectiveness of this across a large diversity of existing cassava genotypes, moreover with high levels of heterozygosity, is not clearly known.

PGRs have been used to induce flowering and fruiting in many crop plants [17, 18, 45]. For example, gibberellic acid (GA_3_) in seedless grape [46], benzyladenine (BA) in *Jatropha curcas* [47], silver thiosulfate (STS) in *Oldenlandia herbacea* [48] and a mixture of GA_3_ + BA + Boric acid in date palm (*Phoenix dactylifera* L.) [49]. Application of PGRs is increasingly becoming important in enhancing flower and fruit set in cassava. Different PGR types have been used in this regard, though with varying degrees of success. For example, indoleacetic acid (IAA), naphthalene acetic acid (NAA) and ascorbic acid [50], Paclobutrazol (PBZ) and potassium nitrate, KNO_3_ [51]. Recent reports on application of STS [26] and BA together with STS [14] show promising results. However, the efficacy of these PGRs on cassava genotypes of Uganda (Table 1) needs verification and/or optimization.

The pruning technique has also been used widely to manipulate flower and fruit production in ornamental and horticultural plants [52]. Increased flowering by pruning has been reported in Pomegranate (*Punica granatum* L.) [25] and *Jasminum sambac* var. Baramasi at different dates [53] and seasons [54]. In [30] and [31] enhanced fruit and seed set in abortive cassava flowers when pruning is applied together with PGRs has been reported. Inability to produce flowers and flower abortion at early branching levels is one of the impediments to cassava hybridization in Uganda. Whether pruning can be used to overcome this challenge has not been investigated. The aim of this study, therefore, was to investigate the effectiveness of red light photoperiod extension in enhancing flowering and fruit set in cassava genotypes of Uganda under field conditions. Additionally, this study sought to assess effects of plant growth regulators and pruning supplementation under red light treatment on flowering. Findings of this study will enable breeders to develop frameworks for integration of photoperiod extension in cassava breeding operations routinely undertaken in crossing nurseries.

## Results

### Impact of extended exposure to red light on forking traits 

**Fig. 1:**
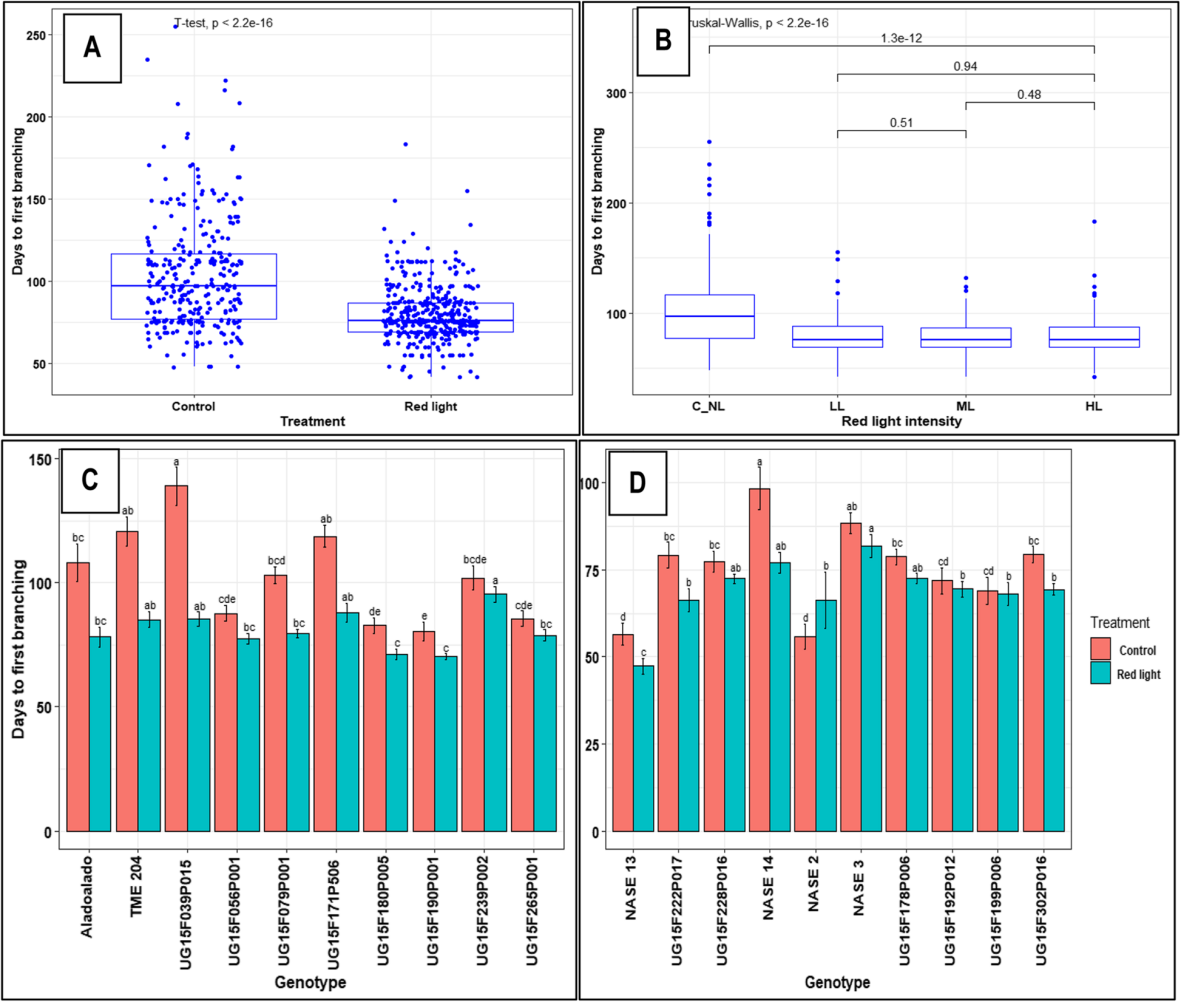
Effect of extended red light exposure and intensities on time to first branching: **A** Days to first branching in controls and RLE treatments for all hard-to-flower genotypes; **B** Days to first branching all hard-to-flower genotypes under different red light intensities; **C** Days to first branching in Hard-to-flower genotypes under control and RLE treatments; **D** Days to first branching in Easy-to-flower genotypes under control and RLE treatments. Data shown are means and SEM obtained from six replications over two growing seasons. Different lower case letters on bars indicate significant differences between genotypes and photoperiod treatment combinations evaluated by Tukey’s HSD test at *P* ≤ 0.05. C_NL = no, LL = low, ML = medium and HL = high red light intensities

Flowering in cassava is preceded by forking or branching of the primary stem. Some genotypes take a long time to form floral forks while others hardly do so. Averaged across all genotypes, RLE and red light at all three intensities resulted in a significant reduction (*P* ≤ 0.001) in time (days) to first forking compared to control (Fig. 1A, B). The hard-to-flower genotypes benefited more than the easy-to-flower ones, though the response was genotype-dependent, with UG15F039P015 (85.4 *vs* 138.9 days), TME 204 (85.1 *vs* 120.5 days), UG15F079P001 (79.5 *vs* 103.0 days) and Aladoalado (78.1 *vs* 108.0 days) being the most responsive (Fig. 1C, D). The different red light intensities (LL, ML and HL) were not significantly different in induction of early forking across genotypes (Fig. 1B). In NASE 2, contrastingly, RLE led to delayed initiation of flowering. Early forking consequently resulted in shorter heights and fewer numbers of nodes at first tier or branching (Table 1), e.g. UG15F039P015 (56.5 *vs* 124.3 cm and 24 *vs* 47 nodes). This was significant even at all light intensities (*P* ≤ 0.001).

**Table 1:** Summary of effects of red light treatment on forking characteristics in hard- and easy-to-flower genotypes

Genotype	Fork (%)		Ht_T1(cm)		Nodes_T1 (Number)		T1_branches (Number)		Tiers (Number)	
	**C_NL**	**RLE**	**C_NL**	**RLE**	**C_NL**	**RLE**	**C_NL**	**RLE**	**C_NL**	**RLE**
**Hard-to-flower genotypes**										
Aladoalado	77.4	100.0	96.4	60.8 bc	47	29 a	3	2b c	2	4 c
TME204	94.3	100.0	129.5	82.2 a	46	32 a	3	3 a	3	4 c
UG15F039P015	79.2	100.0	124.3	56.5 b	47	24 ab	2	2 c	2	5 c
UG15F056P001	96.6	100.0	59.6	51.5 ef	25	22 ef	3	2 bc	6	6 a
UG15F079P001	97.1	100.0	102.5	73.2 b	36	28 bc	3	3 ab	4	5 b
UG15F171P506	87.1	100.0	84.9	57.2 cd	36	26 bcd	3	2 bc	4	3 c
UG15F180P005	100.0	100.0	50.0	41.7 f	23	19 f	3	2 c	6	6 a
UG15F190P001	100.0	100.0	92.9	81.9 b	30	25 de	3	3 a	6	6 a
UG15F239P015	96.3	100.0	55.2	48.2 ef	31	29 cd	3	3 a	5	5 b
UG15F265P001	100.0	100.0	65.0	56.6 de	29	24 def	2	3 bc	5	5 b
**P (Genotype)**	***		***		***		***		***	
**P (Treatment)**	***		***		***		*		***	
**Easy-to-flower genotypes**										
NASE 13	100.0	100.0	54.4	37.2 cd	22	17 c	2	2 c	6	7 a
NASE 14	96.0	100.0	58.6	38.0 cd	30	23 ab	2	2 abc	5	5 cde
NASE 2	100.0	100.0	15.3	17.5 e	8	10 d	3	2 c	6	6 ab
NASE 3	97.0	100.0	67.1	47.3 abc	30	25 a	3	3 a	5	5 bcde
UG15F178P006	100.0	100.0	59.1	47.0 abc	28	23 ab	3	3 ab	6	6 bc
UG15F192P012	100.0	100.0	57.7	40.8 bc	22	18 bc	3	3 bc	6	5 bcde
UG15F199P006	100.0	100.0	29.4	26.6 de	17	16 cd	3	2 c	7	7 a
UG15F222P017	100.0	100.0	55.9	42.5 bc	25	21 abc	3	3 bc	5	5 de
UG15F228P016	100.0	100.0	54.5	52.5 ab	25	24 a	2	2 c	6	5 e
UG15F302P016	100.0	100.0	72.4	57.9 a	24	23 ab	3	3 a	6	6 bcd
**P (Genotype)**	ns		***		***		***		***	
**P (Treatment)**	ns		***		***		***		ns	

*C_NL* control with no red light exposure at night, *RLE* red light exposure; T1_branches = number of branches at first branching; Ht_T1 = stem height at first branching; Nodes_T1 = number of nodes at first branching. Data includes means of 10 hard-to-flower genotypes grown in six replications over two growing seasons, 2019–2020 at Namulonge. Except for ‘Fork’ which was scored continuously up to 12 months after planting, the other variables were scored once at 12 months. Different lower case letters within columns indicate significant differences for averages across C_NL and RLE treatments among genotypes by Tukey’s HSD test at *P* ≤ 0.05. ANOVA tests of significant main effects are indicated: *P* ≤ 0.10 (•), *P* ≤ 0.05 (*), *P* ≤ 0.01 (**), *P* ≤ 0.001 (***)

Compared with control (C_NL), RLE caused forking of the primary stem (100%) in all the late- or non-flowering (hard-to-flower) genotypes. Additionally, RLE caused a significant increase (*P* ≤ 0.001) in the number of tiers in some of the hard-to-flower genotypes, such as UG15F039P015 (5 *vs* 2 levels) as shown in Table 1. Similarly, RLE significantly affected number of first tier branches. The different light intensities, except HL (P = 0.0363), had no significant effect on the number of branching levels.

### Impact of extended red light exposure on flowers, fruit- and seed set

**Fig. 2:**
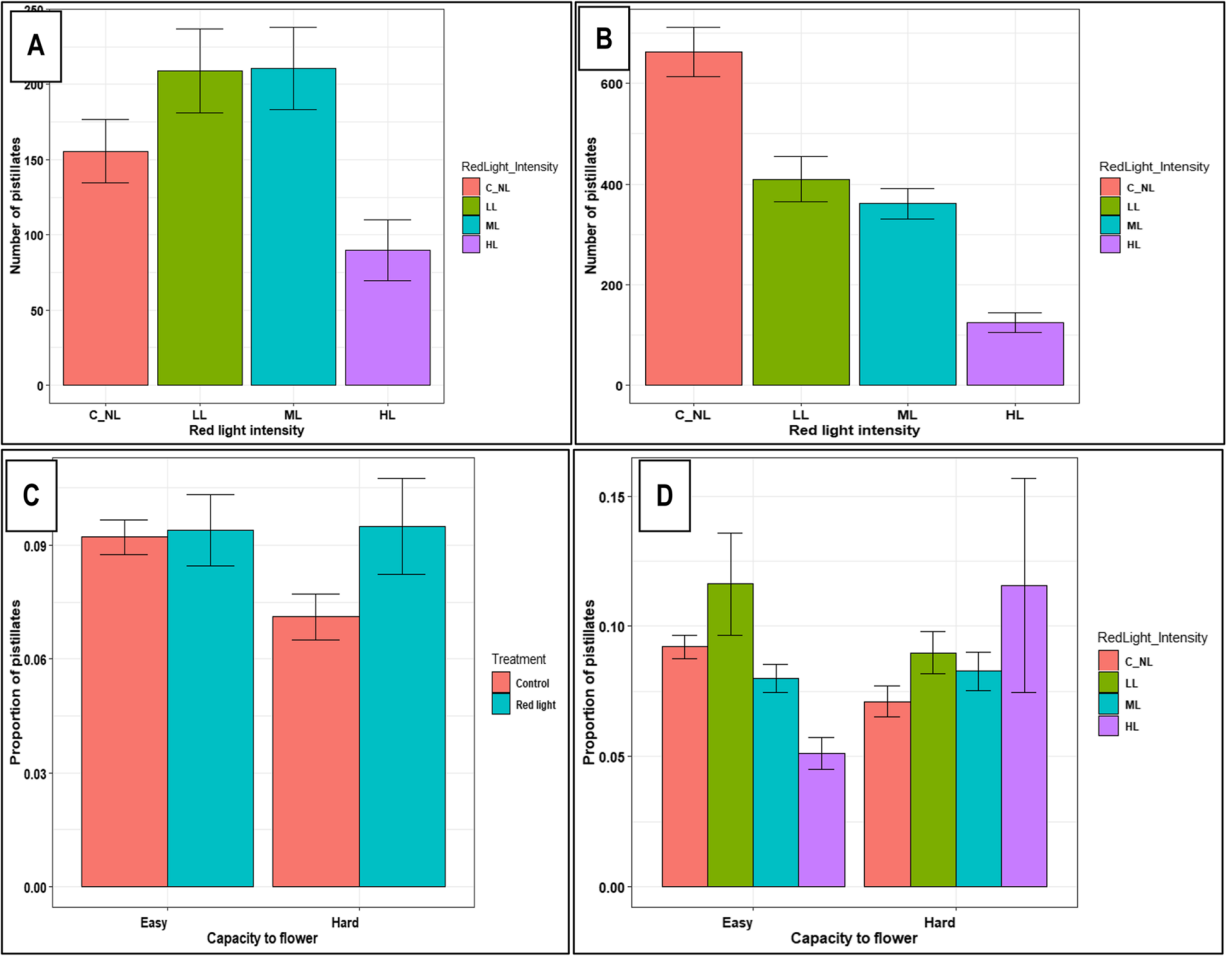
Effect of red light exposure and intensities on number and proportion of pistillates: **A** Pistillate flower numbers in hard-to-flower genotypes in response to RLE treatments of various red light flux densities; **B** Pistillate flower numbers in easy-to-flower genotypes in response to RLE treatments of various red light flux densities; **C** Pistillate flower proportion in easy- and hard-to-flower genotypes under control and RLE treatments (averaged across all flux densities); **D** Pistillate flower proportion in easy-to-flower and hard-to-flower genotypes under control and RLE treatments of various red light flux densities. Data shown are means and SEM obtained from six replications over two growing seasons. Easy = easy-to-flower, and Hard = hard-to-flower cassava genotypes

Among the hard-to-flower genotypes, RLE significantly increased the total number (*P* ≤ 0.001) and proportion (*P* ≤ 0.05) of pistillate flowers except UG15F171P506 and UG15F180P005 (Table 2). While, among the easy-to-flower genotypes, number and proportion of pistillates was only enhanced in one genotype, UG15F222P017 (*P* ≤ 0.01). RLE had a significant effect (*P* ≤ 0.001) on number of pistillates but not on proportion of pistillates among all flowers. Red light intensities, LL and ML had a more stimulatory effect than HL on pistillate flower induction in all the genotypes (Fig. 2A, B). Relative influence of RLE on proportion of pistillate flowers in the hard- and easy-to- flower genotypes is shown in Fig. 2C, D. Figure 2D generally indicates that LL intensity was more stimulatory in increasing the fraction of pistillate flowers in the easy-to-flower genotypes, while in the hard-to-flower genotypes it was HL intensity that was most stimulatory, though different genotypes responded with different magnitudes.

**Table 2:** Effect of red light exposure on number of pistillates, fruits and seeds in Hard-to-flower and Easy-to-flower genotypes

Genotype	Number of pistillates (% of control)	Proportion of pistillates (% of control)	Fruits (% of control)	Seeds (% of control)
**Hard-to-flower**				
Aladoalado	179.2 c	101.5 bc	193.2 c	180.9 b
TME 204	130.5 c	95.6 bc	165.4 c	127.9 b
UG15F039P015	463.8 c	305.9 c	902.9 c	313.2 b
UG15F056P001	147.9 c	124.6 bc	279.0 c	161.2 b
UG15F079P001	205.6 c	158.3 bc	411.0 c	329.5 b
UG15F171P506	66.1 c	87.3 bc	71.2 c	98.9 b
UG15F180P005	60.8 b	110.6 b	78.3 b	83.5 b
UG15F190P001	106.3 a	134.1 a	135.9 a	100.3 a
UG15F239P002	155.5 c	125.0 bc	1143.0 c	356.5 b
UG15F265P001	952.9 c	427.3 c	9276.9 c	1768 b
***P*-value (Genotype)**	***	***	***	***
***P*-value (Treatment)**	***	*	*	*
**Easy-to-flower**				
NASE 13	54.6 c	118.9 b	69.4 d	110.1 c
NASE 14	44.4 bc	88.8 b	50.9b cd	97.6 bc
NASE 2	66.3 bc	71.3 ab	170.3 d	220.8 bc
NASE 3	53.3 a	90.8 a	71.7 a	108.7 a
UG15F178P006	40.0 a	95.2 b	59.7 a	91.3 bc
UG15F192P012	37.8 bc	80.6 b	29.0 bc	87.0 bc
UG15F199P006	57.3 c	84.8 b	57.2 bcd	83.5 bc
UG15F222P017	116.4 c	122.0 b	132.4 cd	137.1 abc
UG15F228P016	59.8 c	85.1 c	85.7 d	95.8 c
UG15F302P016	75.1 ab	187.3 ab	66.4 b	81.2 ab
***P*-value (Genotype)**	***	***	***	***
***P*-value (Treatment)**	***	ns	***	ns

Data includes means of genotypes grown in six replications over two growing seasons. Percentages of control were obtained by dividing means of genotypes under red light treatment with means under control. Proportions of pistillates were obtained by dividing number of pistillate flowers with staminate flowers. Different lower case letters within columns indicate significant differences among genotypes by Tukey’s HSD test at *P* ≤ 0.05. *P* ≤ 0.10 (•), *P* ≤ 0.05 (*), *P* ≤ 0.01 (**), *P* ≤ 0.001 (***), *ns* not significant

**Fig. 3:**
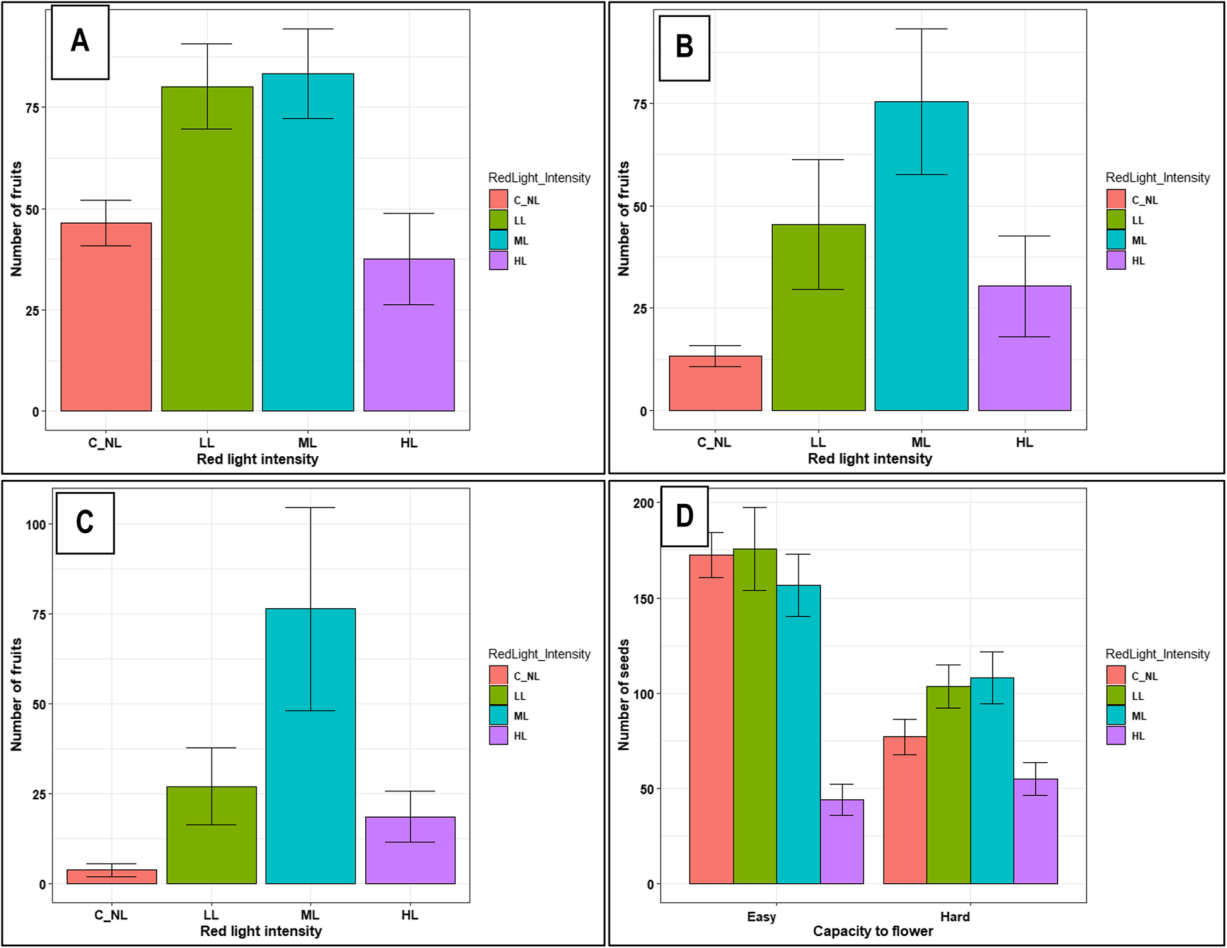
Effect of extended red light intensities on fruit and seed set: **A** Number of fruit set in all genotypes under different red light flux densities; **B** number of fruit set in the genotype UG15F079P001 under different red light flux densities; and (**C**) number of fruit set in the genotype UG15F039P015 under different red light flux densities; **D** Number of seed set in easy- and hard-to-flower genotypes under different red light flux densities. Data shown are means and SEM obtained from six replications over two growing seasons

Among genotypes, 80% of the hard-to-flower, compared to 20% of the easy-to-flower genotypes, had enhanced fruit and seed set (Table 2). LL and ML intensities caused the highest fruit set (Fig. 3A). UG15F079P001 (Fig. 3B) and UG15F039P015 (Fig. 3C) were among the genotypes with greatly improved fruit set. Comparative effects of different red light intensities on seed set in the hard- and easy-to-flower genotypes are shown in Fig. 3D.

**Fig. 4:**
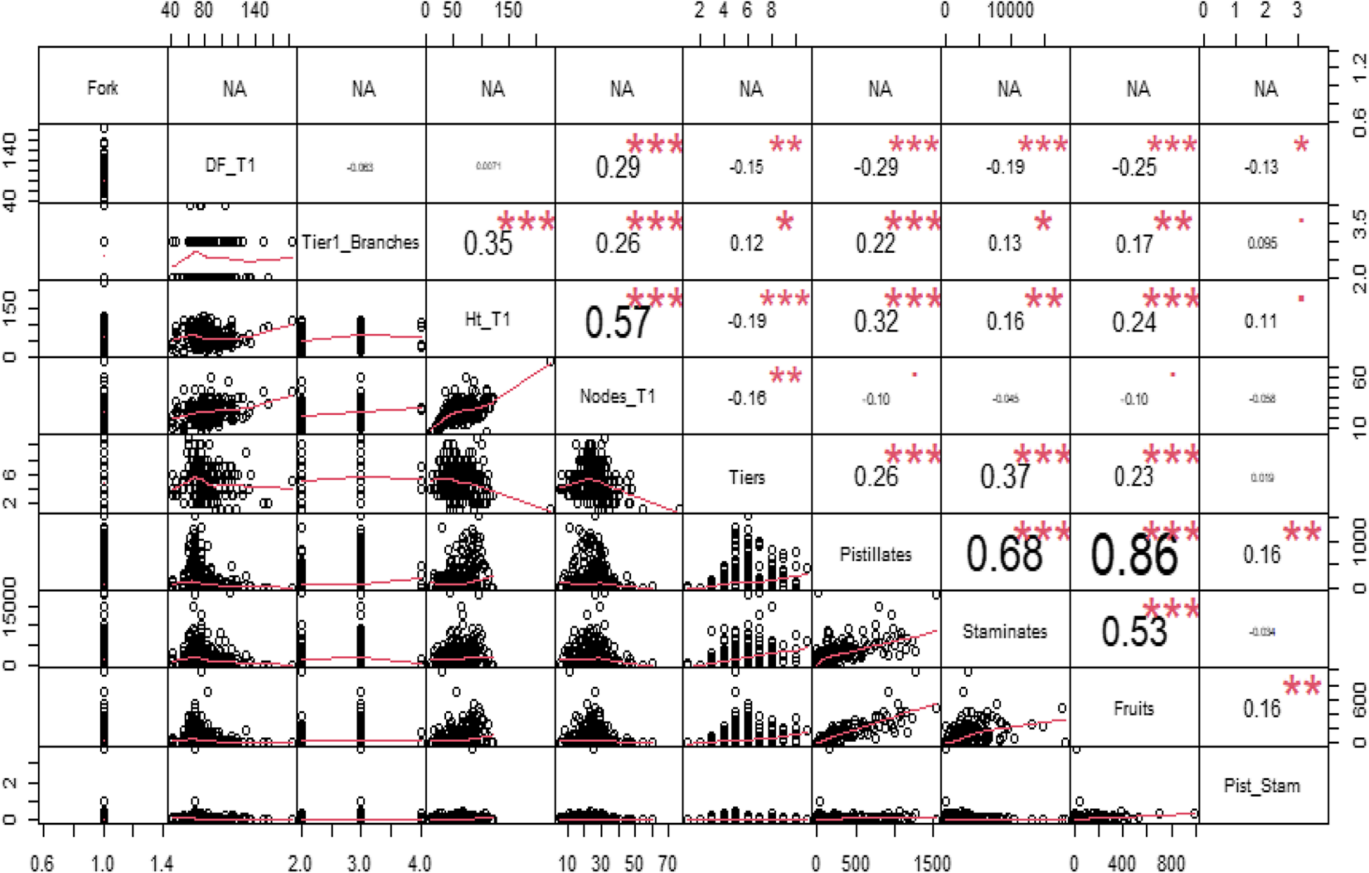
Correlation results based on the Pearson coefficient for parameters measured in hard-to-flower cassava genotypes under RLE. Values represent the correlation coefficients, and asterisks indicate significance (*P* ≤ *0.10 (•), P* ≤ 0.05 (*), P ≤ 0.01 (**), P ≤ 0.001 (***)). T1_branches = number of branches at first tier; DF_T1 = days to first forking; Ht_T1 = stem height at first tier; Nodes_T1 = number of nodes at first tier; Pist_Stam = proportion of pistillates

For both categories of treated genotypes used in the study, strong relationships (r = 0.86, P ≤ 0.001) were observed between pistillates and fruits as compared to controls. Correlation values increased to 0.86, from 0.67 and 0.80 in easy-to-flower and hard-to-flower genotypes respectively. In the hard-to-flower types **(**Fig. 4), the correlations were moderate between height at tier 1 (Ht_T1) and nodes (Nodes_T1) (*r* = 0.57), pistillates and staminates (*r* = 0.68) and between staminates and fruits (*r* = 0.53) and nodes (*r* = 0.58). Forking did not show any correlations.

### Effect of supplementing red light extension with plant growth regulators and pruning on flowering and fruit set 

**Fig. 5:**
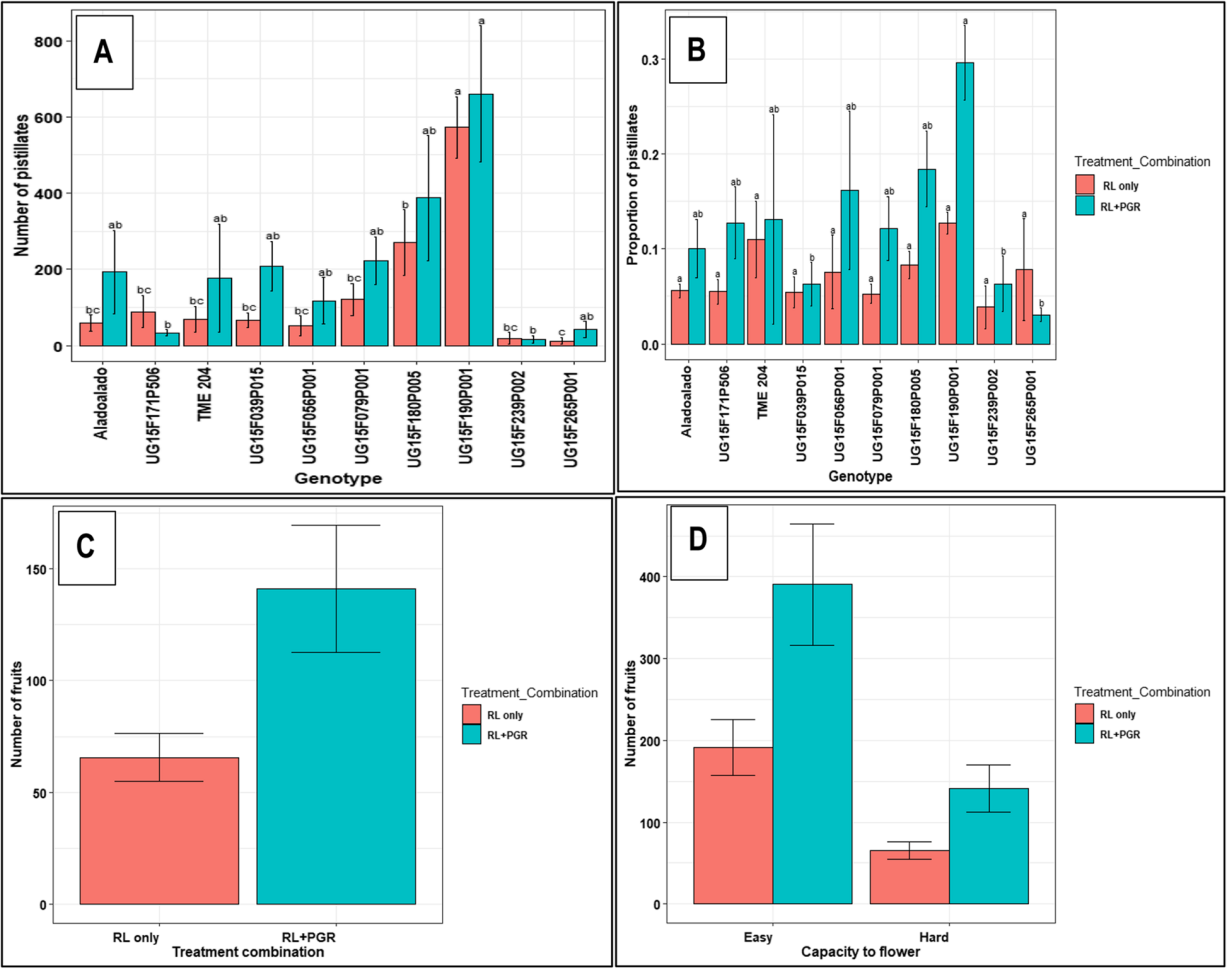
Effect of supplementing red light photoperiod extension with PGR application on flowering and fruit set: **A** number of pistillate flowers in hard-to-flower genotypes in response to RLE only or RLE + PGR; **B** proportion of pistillate flowers in hard-to-flower genotypes in response to RLE only or RLE + PGR; **C** fruit set and survival in response to RLE only or RLE + PGR in hard-to-flower genotypes; and (**D**) comparison of fruit set and survival in easy- and hard-to-flower genotypes under RLE only or RLE + PGR. RL = red light extension; RL + PGR = red light + plant growth regulators; Easy = easy-to-flower; and Hard = hard-to-flower cassava genotypes. Different lower case letters on bars indicate significant differences among genotype X treatment combinations by Tukey’s HSD test at *P* ≤ 0.05

Generally, supplementation of RLE with PGR (STS + BA) significantly enhanced number and proportion of pistillate flowers (*P* ≤ 0.05) (Fig. 5A, B) as well as fruits (*P* ≤ 0.001) (Fig. 5C) compared to RLE alone. This supplementation enhanced fruit set and survival by more or less same proportion in the easy-to-flower and the hard-to-flower genotypes (Fig. 5D). Overall, fruit set and survival was enhanced in over 90% of the genotypes used in this study.

**Fig. 6:**
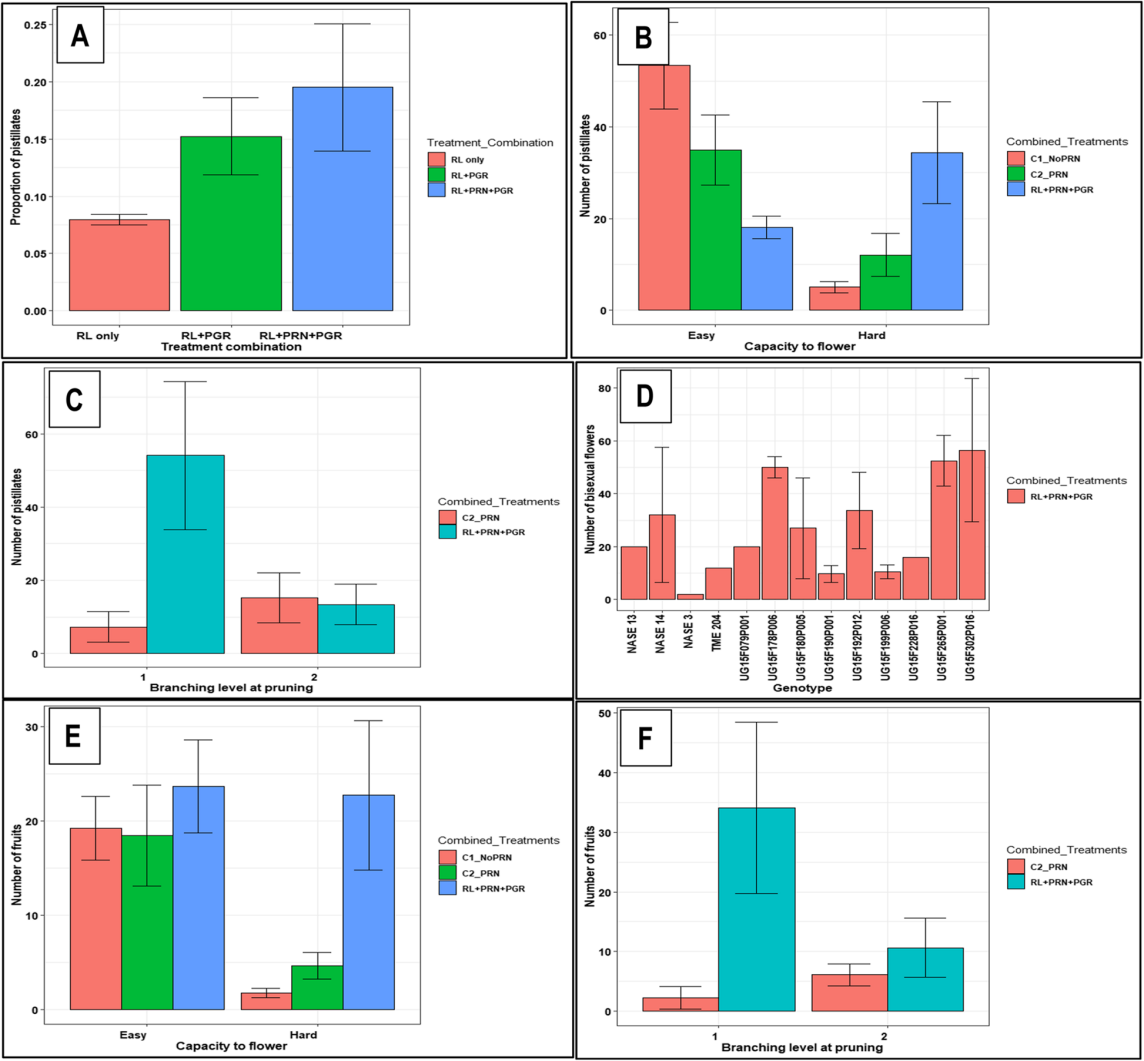
Effect of combining red light photoperiod extension with pruning and PGR applications on flowering and fruit set at first and second branching levels: **A** proportion of pistillate flowers in response to RLE only, versus RLE in combination with PGR or PGR + pruning in hard- and easy-to-flower genotypes; **B** number of pistillate flowers in response to control versus the combined RLE + pruning + PGR treatments in hard-to-flower and easy-to-flower genotypes; **C** number of pistillate flower s in plants pruned at the first versus second branching level in controls versus the combined treatment RLE + PGR in hard-to-flower genotypes; **D** number of bisexual flowers in response to the combined treatment of RLE + PRN + PGR in genotypes; **E** fruit set and survival in easy- versus hard-to-flower genotypes under treatments with no-RLE/noPRN, no-RLE/ + PRN, or RLE + PRN + PGR; **F** number of fruit set and survival in plants pruned at the first versus second branching level in controls versus the combination treatment RLE + PGR in hard-to-flower genotypes. Data shown are means and SEM obtained from six replications over two growing seasons. Pistillate_Staminate = ratio of pistillate to staminate flowers; RL = red light extension; RL + PGR = red light extension + plant growth regulator; Easy = easy-to-flower; Hard = hard-to-flower cassava genotypes; C1_NoPRN = control (no RLE) without pruning; C2_PRN = control (no RLE) with pruning; PRN = pruning

**Fig. 7:**
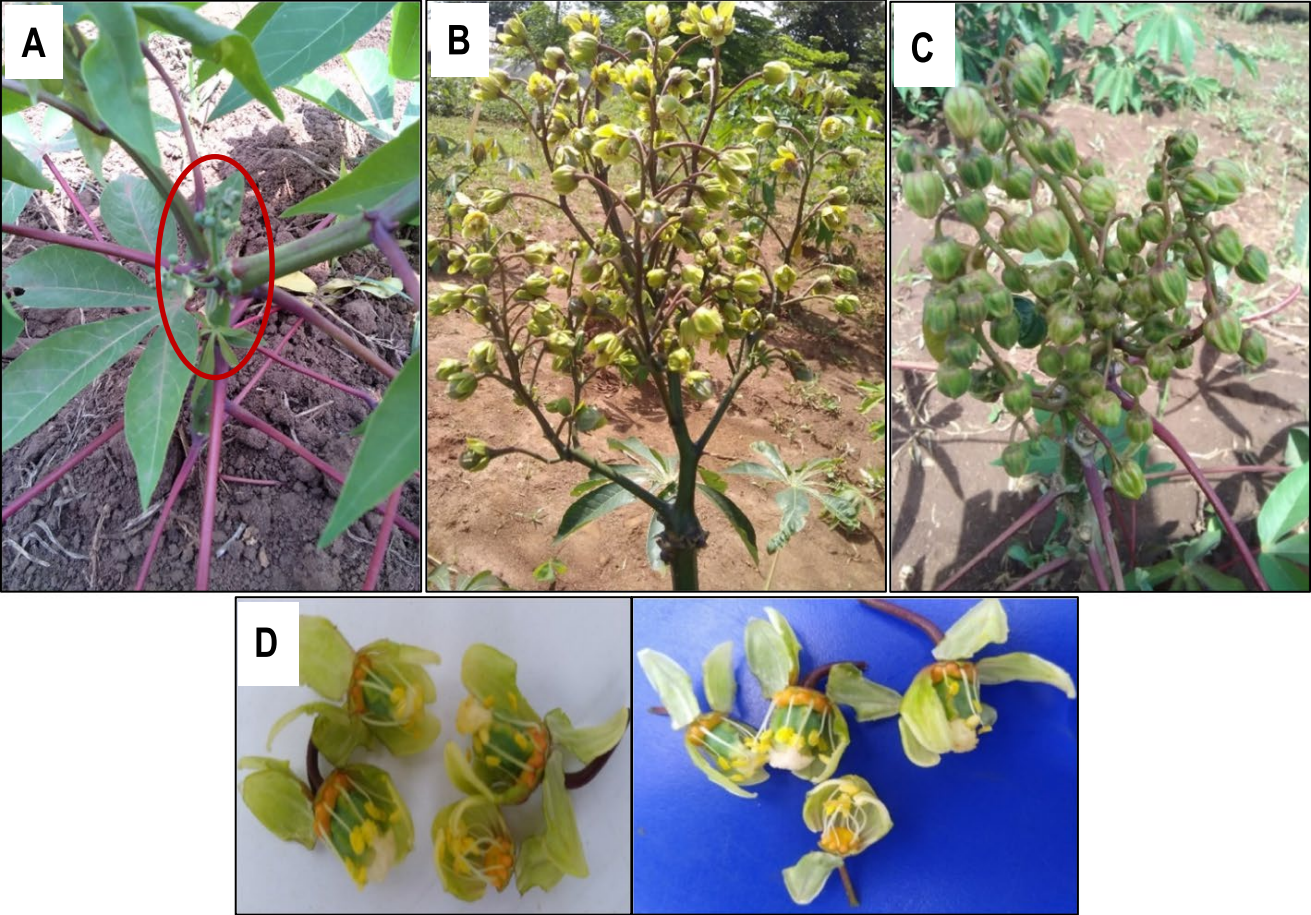
Prolific flowering and feminization in cassava flowers at first branching following application of pruning and PGR under red light exposures: **A** Control plant (UG15F199P006) with a poorly developed and aborting inflorescence (encircled in red); **B** UG15F265P001 (a hard-to-flower genotype) pruned at first branching level; **C** UG15F199P006 (an easy-to-flower genotype); **D** Hermaphrodite flowers

Pruning applied together with PGR significantly increased the number and proportion of pistillate flowers (*P* < 0.001) compared to pruned and unpruned controls in the genotypes subjected to RLE (Fig. 6A). However, the increase in pistillates was mainly significant in the hard-to-flower genotypes and not in the easy-to-flower ones (Fig. 6B). Pistillate flower induction was maximal at flowering level one (first branching) (Fig. 6C). Combined pruning and PGR application also induced feminization of staminate flowers, leading to formation of bisexual flowers (Fig. 6D). This phenomenon is clearly shown in Fig. 7. Fruit set and survival significantly increased under pruning and PGR treatments (Fig. 6E) at first branching (Fig. 6F). However, it was noticed that number of leaves at first flowering event in some genotypes was not enough and would soon become too old to sustain the set fruits to maturity. Pruning plants under no RLE was generally not beneficial (Fig. 6E).

## Discussion

Objectives of this study were to investigate the extent to which exposure to red light throughout the night to create a long-day photoperiod would induce earlier flowering, and the extent to which supplementation of plant growth regulators and pruning would overcome the challenges of a) no or delayed flowering, b) sparse flower production, c) low proportion of pistillate flowers, and d) poor fruit set in cassava. Results of this study showed that red light photoperiod extension induced earlier flowering and enhanced flower formation and fruit set in cassava genotypes of Uganda grown under field conditions, where cassava breeding requires reliable, synchronous, and prolific flowering.

Treatment with RLE resulted in reduction in height and number of nodes to-forking/flowering as well as increased number of branching levels during the crop season. These architectural changes favored shortened flowering time, by about two months and increased flowering events, by about three. This is consistent with the findings of contemporary studies on cassava by [33, 34] in response to RLE, and is in corroboration with the findings by [55] which indicated shortened flowering time in *Arabidopsis thaliana* when subjected to night- (light-) breaks using red light. In [56], a reduction in shoot length of *Petunia* × *hybrida* when exposed to red light was also reported. Comparatively, days to flowering and number of nodes were reduced in *Dianthus* when subjected to night breaks [57].

Light as an important environmental signal, interacts with endogenous signals in plants to induce photomorphogenesis [58]. Phytochromes, one of the light receptors in plants, exists in two interconvertible forms: an inactive red light-absorbing (Pr) and active far red light-absorbing (Pfr) forms [59]. They are known to be involved in photomorphogenic changes that culminate in flowering [40]. The peak of red light LED lamps used in this study was at 660 nm, which is the absorption maximum for Pr. Therefore, consistent with the findings of [60], a night-long exposure to red light in this study may have induced a photo-conversion of a high proportion of Pr to the Pfr form, which promoted the observed “photomorphogenic” traits. Regulation of flowering in plants is multifaceted, involving an interaction of factors such as photoperiod, light quality, and gibberellins (GA) [61]. Environmental signaling through prolonged exposure to light is known to induce flowering in long day plants such as *Arabidopsis.* The reduction of plant height and number of nodes could have been due to an inhibitory effect of RLE on stem elongation which is reversed by far-red light and as such recognized as the shade avoidance response. Comparable effects were observed by [62] in poinsettia plants (*Euphorbia pulcherrima* Willd. ex Klotzsch). In addition, by stimulating earlier flowering, RLE creates fork-type branches which shortens plant height (Table 2).

Delayed and unsynchronized flowering as well as poor flower and fruit set are among the impediments to crossing and to the goal of hastened or speed breeding in cassava [16]. So, the identification of treatments that elicit early flowering and increased branching events leading to more flower production are important findings in this study. Manipulation of photoperiod by application of red light opens up new perspectives in enhancing breeders’ efforts towards successful crossing and/or hybridizations in cassava breeding programs, through synchronized and greater flower production. Though the effect of RLE in this regard was marginal, among the early or easy-to-flower genotypes, a good number of the late or hard-to-flower were highly responsive. So, deployment of this technology in crossing nurseries involving late-flowering genotypes will allow early fruit and seed set.

RLE substantially increased the number and proportion of pistillate flowers, fruit and seeds in the hard-to-flower genotypes compared to the easy-to-flower ones and/or the controls in which pistillate formation was suppressed **(**Fig. 2 and 3). Studies documenting effects of red light photoperiod extension on flower and fruit numbers under field conditions in the tropics are limited. Nonetheless, some studies (involving night interruption photoperiod treatments) have however, observed quantitative flowering responses in herbaceous perennials [63] and in *Dianthus* using fluorescent lamps emitting high red (R) light and little far-red (FR) light [57]. Similarly, continuous lighting (high R: FR) effectively promoted flower numbers in plants of carnation (*Dianthus*), a long day plant [64]. Whereas the improved numbers in the hard-to-flower genotypes could be attributed to a stimulatory effect of RLE, the high pistillate numbers among the controls in the easy-to-flower genotypes was due to their inherent capacity to flower. Variation among responsive genotypes could be attributed to genotypic differences in genes that are involved in photoperiod and hormone systems that are involved in regulating flowering.

Correlation analyses showed varying degrees of relationships between flowering and/or seed set attributes measured in this study. In particular, there was enhanced strong positive relationship between pistillates and fruits (*r* = 0.86). Though not strictly comparable, these results are corroborated by [65] who also showed positive significant correlation between flowers and fruits in tomato (*Solanum lycopersicum* L.). This is so biologically meaningful in that fruits arise from flowers. So, the application of red light treatment in combination with pruning and PGRs had an additive effect that resulted in the observed relationships. Thus, this indicates that application of red light holds great potential towards improving flowering and fruit set.

In the present study, supplemental treatments with PGR and pruning quantitatively increased pistillates and fruits in both hard- and easy-to-flower genotypes. A previous study demonstrated increased flower numbers when pruning was applied with or without BA and STS compared to the control [14]. Similarly, pruning under extended day length increased fruit and seed set [31]. Also, consistent with the findings of this study is the fact that pruning was more effective in enhancing flowering at first level of forking than the second (Fig. 6C). Increased flowering and fruiting using BA was also reported in *Jatropha curcas* [47] and horticultural crops such as date palm (*Phoenix dactylifera* L.) [49]. Therefore, PGR and pruning applications in this study, enhanced fruit set by preventing abortion of flowers and young fruits. Pruning is believed to strengthen apical dominance of the terminal inflorescence hence preventing abortions commonly exhibited at first branching. STS, an anti-ethylene PGR, works to prolong the life and freshness of flowers [66]. A similar effect is believed to have occurred in this study, preventing premature flower and fruit abortions. Similarly [26], reported that STS increased flower production and longevity in cassava under glass house conditions. Regarding application of BA in this study, it may have stimulated pistillate flower formation and increased fruit set. This effect has also been reported by [14] in cassava and is corroborated by [67]. Thus, these results indicate that effective enhancement of flowering and fruit set under RLE can best be maximized with PGR and/or pruning supplementation.

Feminization of staminate flowers due to BA (and STS) application with or without pruning was observed in this study and was reported among the germplasms used by [14] and [31]. Normally, cassava flowers exhibit unisexuality, being either pistillate or staminate flowers [9, 68]. Feminization, caused by BA treatments has also been reported in *Jatropha curcas* [67]. Since one of the limiting factors in cassava crossing nurseries is a lower ratio of female: male flowers, feminization could offer a solution to this challenge. The actual mechanism of feminization is not known, but could be due to an interaction between the exogenous signals such as applied PGR and floral integrator or floral organ-identity genes which are known to control differentiation of floral organs during flower development.

Findings of this study indicated that low- to medium red light intensities had more stimulatory effects on most of the parameters measured compared to high light intensity. This observation is in agreement with findings of [33] who reported that a minimum light quantity of about 0.02 µmol m^−2^ s^−1^ is just enough to elicit a flowering response. Runkle and Heins [69] contend that light intensity required for effective photoperiodic lighting is typically very low. In fact, low-intensity lighting has been reported to be more effective at promoting flowering in long day plants, especially if it contains both R and FR [69].

## Conclusion

Results of this study show that RLE is effective in inducing and/or enhancing flowering in cassava through shortening flowering time, reduction of height and number of nodes at first branching, increasing flowering or branching events, number and proportion of pistillate flowers. Hard-to-flower genotypes such as Aladoalado, TME204, UG15F039P015, UG15F079P001 and UG15F171P506 were more responsive in forking and earliness to flower, which was reflected by fewer days to first branching, reduced height and number of nodes at first branching. RLE also increased number of branching levels, proportion of pistillate flowers as well as their correlation with fruit set. LL to ML intensities were the optimal stimulatory levels of red light. Enhanced flowering following exposure to red light indicates that cassava is a long day plant. This is corroborated by findings of a recent study by [70] and previous observations made on the crop under natural light conditions by [44].

Supplementing with PGR (BA and STS) and pruning applications substantially enhanced flowering and fruit set through minimizing flower abortions. These applications caused feminization of staminate flowers that led to increased proportion of pistillate flowers as well as number of fruits. Pruning was particularly more effective at the first level of branching, enabling fruit formation in the genotypes that never set fruit at lower branching levels. The current findings provide insight into solutions that can be used to overcome bottlenecks to cassava hybridization.

## Materials and methods

### Location and field conditions

**Fig. 8:**
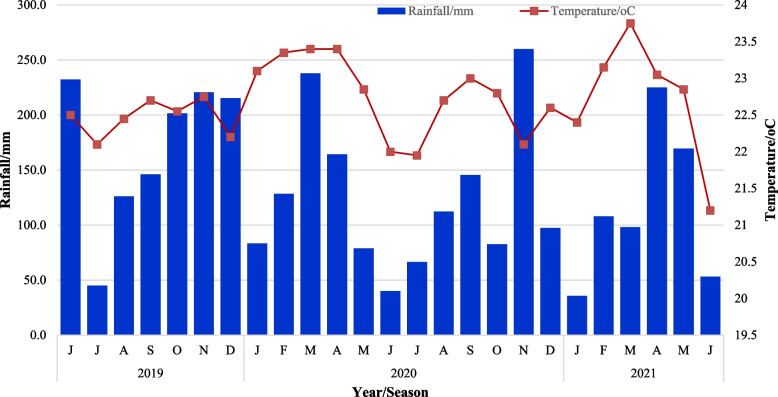
Average monthly rainfall and temperature during the study period, June 2019 to June 2021

The study was conducted under field conditions through two growing seasons (2019/2020 and 2020/2021, from June to June of each season) at the National Crops Resources Research Institute (NaCRRI), Namulonge in central Uganda. NaCRRI hosts the national cassava breeding program, where most crossings and hybridizations are conducted. Namulonge (32° 34’E, 0° 32’N) is located at 1200 m above sea level with a natural photoperiod of about 12 h which is fairly uniform throughout the year. Historically, it is characterized by an average annual rainfall of approximately 1300 mm, average annual temperature of 22 °C, and annual minimum and maximum temperature of 16 and 28 °C, respectively. The soils are dark, reddish-brown, sandy-loam, orthic ferralsols with a pH range of 5.5–6.2. The data on average rainfall and temperature conditions that prevailed during this study **(**Fig. 8) were obtained from the Namulonge meteorological station.

### Plant materials

A total of 20 cassava genotypes with two major contrasting flowering behaviors were selected for this study, 10 late- (flowering at over five months after planting) or non-flowering (referred to as “Hard”) and 10 early- or moderately flowering (at two to four months after planting) (referred to as “Easy”) (Table 3). These materials were selected from cycle one (C1) population of genomic selection studies (GS) developed at NaCRRI. Briefly, the C1 clones were derived from recurrent crosses and selections among the best a hundred C0 clones selected through GS.

**Table 3:** Selection of study genotypes

No	Genotype	Genotype status	Onset of flowering	Capacity to flower
1	NASE 2	Elite variety	Early	Easy
2	NASE 3	Elite variety	Early	Easy
3	NASE 13	Elite variety	Early	Easy
4	NASE 14	Elite variety	Moderately early	Easy
5	UG15F178P006	Breeding line	Early	Easy
6	UG15F199P006	Breeding line	Early	Easy
7	UG15F228P016	Breeding line	Early	Easy
8	UG15F192P012	Breeding line	Early	Easy
9	UG15F222P017	Breeding line	Moderately early	Easy
10	UG15F302P016	Breeding line	Moderately early	Easy
11	TME 204	Elite variety	Non/very late	Hard
12	Aladoalado	Land race	Late	Hard
13	UG15F265P001	Breeding line	Non/very late	Hard
14	UG15F171P506	Breeding line	Late	Hard
15	UG15F190P001	Breeding line	Late	Hard
16	UG15F180P005	Breeding line	Late	Hard
17	UG15F039P015	Breeding line	Non/very late	Hard
18	UG15F239P002	Breeding line	Non/very late	Hard
19	UG15F056P001	Breeding line	Late	Hard
20	UG15F079P001	Breeding line	Non/very late	Hard

### Field establishment and management

**Fig. 9:**
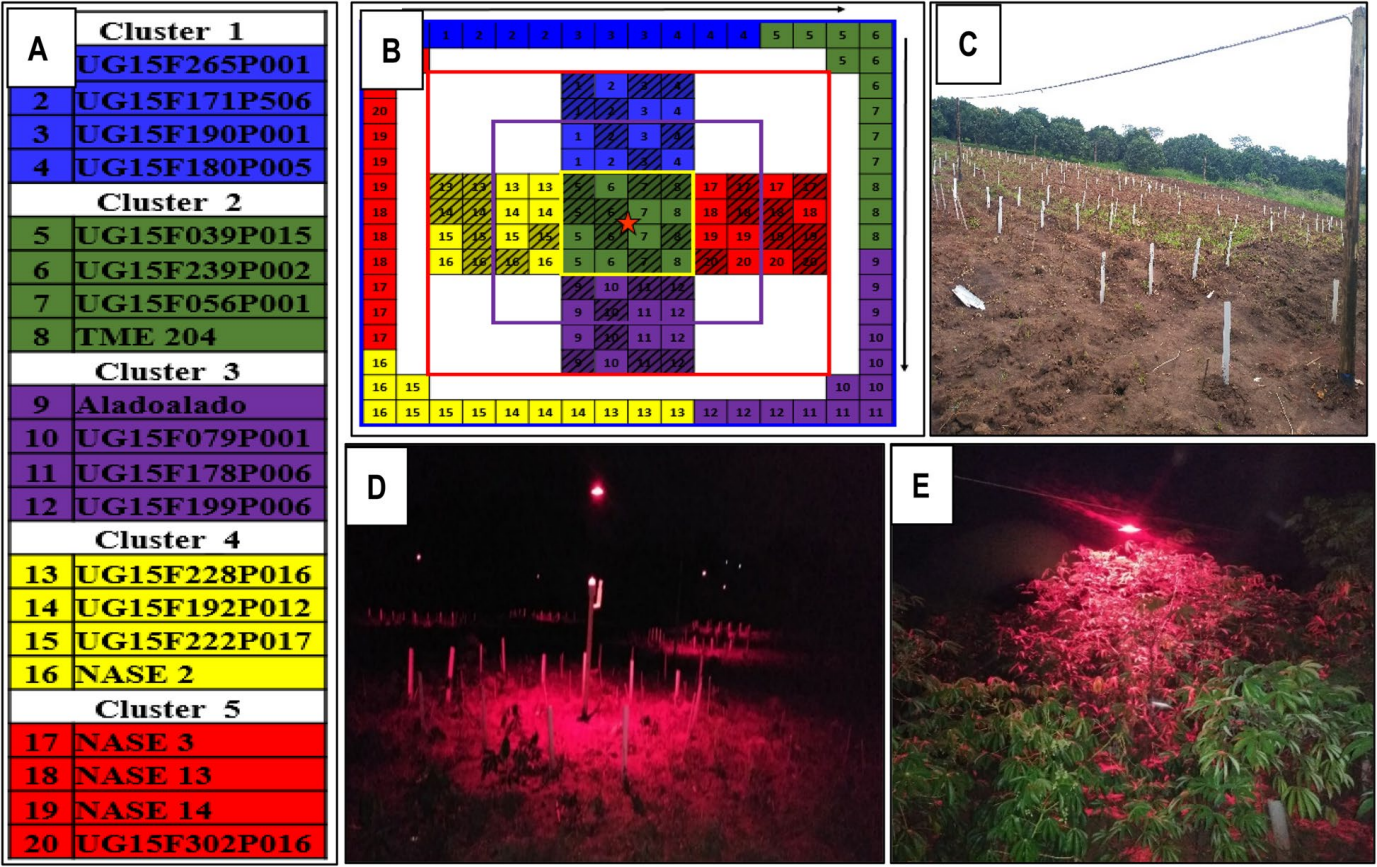
Block layout for red light treatment plus pruning and PGR supplements: **A** Clusters of genotypes; **B** Example of the layout for one of the six replicate blocks, illustrating light intensities, genotype clusters and treatments. The central boxed area, demarked with a yellow box, represents the HL treatment; the region demarked with a purple box is the ML treatment; the region demarked by a red box is the LL treatment; and the periphery is the C_NL treatment. Shaded boxes indicate plants pre-selected for supplements of PGRs and pruning applications; **C** Field layout; (**D** & **E**) Red light treatment exposures (RLE) at early plant stage and when plants had advanced in age

Land, previously used for sweet potato crossing nurseries, was tilled and marked into six experimental blocks of 16 × 16 m. Red-light system was setup using 50W light emitting diode (LED) lamps with red LEDs (model 5–10 × 5w, China) (illumination range 640-660 nm) and with reflectors (339 × 350 mm, model ISL-RFGB, CCS Inc., China) as sources of red light for extension of the photoperiod during the night. One lamp was placed horizontally at 3 m above the ground in the center of each block to cast red light over the plants **(**Fig. 9**)**. Due to the restriction imposed by the red-light system installation, the study genotypes were grouped into five clusters of four each for easy and systematic allocation of the genotypes under the red light treatments **(**Fig. 9A**)**. Assignment of genotypes to a cluster was done randomly. There were six main blocks and the clusters were laid out in a crisscross pattern in the center, with plots, of four plants each, arranged centrifugally in each block. Clusters were assigned to locations randomly such that each cluster was replicated in each red light treatment at least two times over the two growing seasons of the experiment. The control plants were planted peripherally around each block (Fig. 9B). Stem cuttings (~ 25 cm long each) were planted in holes (horizontally) with a spacing of 1 m between plants and rows in each block. Each block served as a replication to address the genetic variation among the study genotypes. Fields were kept free of weeds by hand-hoe weeding and no fertilizers or supplements were added to the soil.

### Red light treatments and photoperiod extension

Red light exposure (RLE) was commenced soon after complete sprouting/germination (about 14 days after planting). Thereafter, daily RLE, was turned on and off automatically at dusk and dawn respectively thus illuminating the plants throughout the night. The plants within a radius of 2 m from center of block were regarded to be in a region of high red light (HL) intensity, ~ 1.0–1.5 PFD (Photo Flux Density of wavelengths 400–700 nm) in µmol m^−2^ s^−1^, a radius of 4 m were in a region of medium light (ML) intensity (0.5–1.0 PFD in µmol m^−2^ s^−1^) and those within a radius of 6 m were in a region of low light (LL) intensity (≤ 0.5PFD in µmol m^−2^ s^−1^). All of the RLE treatments were of dim light relative to typical mid-day solar flux densities of about 2000 µmol m^−2^ s^−1^. Meanwhile, the plants at a radius of 8 m, were in a region of no light (NL), i.e. total darkness (0PFD in µmol m^−2^ s^−1^) and these were used as control (C_NL) (Fig. 9B). Light intensity was measured with a Licor quantum sensor (model LI-190; Lincoln, Nebraska, USA) at distances increasing by increments of 50 cm from 50 to 600 cm from the red LED lamp. This allowed for calculation of light intensity received by plants at increasing distances from the lamp. The intensity for individual plants was spot checked in the field. This experiment was terminated in June, 2021.

### Supplementation with plant growth regulators

Plant growth regulator (PGR) treatments were added as supplements to a few plants subjected to RLE **(**Fig. 9B) in order to provide insight on the possibility of additive effects of combined RLE and PGRs. In this case, one plant per plot was randomly pre-selected for PGR treatment in three blocks (block 1–3), each block acting as a replicate. Two PGRs, STS and BA, which came through as most effective candidates following screening of several PGRs for their effect on cassava flowering [71, 72] were used. Efforts to optimize these PGRs were undertaken in a study by [14]. As such, in this study we chose to undertake (on small-scale) validation of the effectiveness of the STS and BA when used in combination on plants under RLE. Thus, 0.5 mM 6-benzyl adenine (BA) and 4 mM silver thiosulphate (STS) were used. The STS solution was prepared following a modification of the method previously described and optimized by [26]. In this case, 1 part of 0.1 M silver nitrate (AgNO_3_) (Sigma-Aldrich, USA) was added drop-wise to four parts of 0.1 M sodium thiosulfate (Na_2_S_2_O_3_) (Sigma-Aldrich, USA) and diluted with distilled water to the desired concentrations and volumes. The BA solution was prepared by diluting a 6.38 ml (v/v) BA (Sigma-Aldrich, USA or Duchefa Biochemie, Netherlands) stock (1.765 g/100 ml) with distilled water to 1L of solution.

The BA was applied using a hand sprayer at a 7-day interval. In this case, ~ 2.5 ml of solution was directed to 3–5 youngest immature leaves (shoot tip) and inflorescence until just run-off. BA application targeted on the inflorescences was continued until about 14 days after anthesis. Meanwhile STS was administered by sucking through leaf petioles at a 14-day interval as described by [14]. All the treatments for the whole experiment were commenced at the earliest notice of forking in any one genotype, and this routine treatment was continued up to the fourth tier/level of branching, 5 to 8 months after planting, as this varied with genotype.

### Effect of pruning under red light treatment

Pruning and PGRs (BA and STS) treatments were applied on plants under RLE to gain an insight into whether there is any additive effect on flowering and fruit set enhancement using the cassava genotypes in Uganda. This motivation was strengthened by reports from contemporary studies by [31] and [14] which showed that applying pruning on young branches in combination with BA induced flower formation, prevented flower abortion and increased seed–set in cassava germplasms at CIAT (International Center for Tropical Agriculture), Colombia and IITA (International Institute of Tropical Agriculture), Ibadan, Nigeria. In this experiment, one plant per plot (block 1 to 3) was randomly preselected for the combined treatments. Each block served as a replicate. After the stakes had fully sprouted (about 3 weeks after planting), weekly scouting was done to detect initiation of fork‐type branching on the shoot apices. This was noticed by a slight swelling in the width of the apical meristem due to emergence of axillary branches. In the no-red-light controls, pruning (or not pruned) treatments were applied to plants in three replicates. In plants receiving RLE, pruning (or not pruned) treatments were applied to plants that either received PGRs versus those not receiving PGRs (four treatment combinations, each with 3 replicates). Pruning was done by carefully cutting off the new young axillary branches using a sharp razor or surgical blade as described by [31] at the first or second branching levels. BA and STS were applied on the pruned plants as described in Sect. 2.5 above until 3–4 weeks after pruning, when the developing fruits were strong and healthy enough not to abort. Occasionally, false branches below the developed inflorescence after pruning were surgically removed.

### Data collection 

Commencing at about three weeks after planting, weekly assessment of individual plants was done until the fourth branching level and data were recorded on the following parameters: fork of the main stem, scored as “1” for forked or “0” for non-forked; days to first tier of forking/branching (DF_T1), number of female (pistillates) or male (staminates) flowers, fruits and seeds at each tier of flowering/branching. Additionally, forking habit (Tier1_Branches), height (Ht_T1) measured using a meter rule and number of nodes (Nodes_T1) at first fork and number of forking levels (Tiers) were measured and recorded once at 12MAP (months after planting). Mature and ripe fruits were enclosed in muslin bags before drying to avoid loss of seeds due to their inherent explosive nature of dehiscence of dry fruits. Up to three sprouted stems per plant were considered where more than one bud from the planted cutting had sprouted. All data were electronically collected on tablets (model: Lenovo TB-8504F, China) using a Field Book application [73].

### Statistical analysis

Data collected over two seasons were collated, and then disaggregated into components of ‘Hard-’ and ‘Easy-to-flower’ and ‘Combined treatments’ and analyzed separately using statistical models and packages built in [74]. Main stem fork data were categorical (scored as “1” for forked or “0” for non-forked) and thus were analyzed using generalized linear model (glm) by applying a logistic regression model. All count data were analyzed with generalized linear mixed models (GLMM). Count data that were equidispersed (variance = mean) such as DF_T1 and Nodes_T1 were analyzed by applying Poisson model; under-dispersed data (variance < mean) such as Tier1_Branches and Tiers were analyzed using a generalized Poisson model while the over-dispersed count data (variance > mean) such as pistillates, staminates, fruits and seeds were analyzed by applying a negative binomial distribution model, as recommended for data of this kind [75–77].The over dispersion in this case was caused by the zero counts that were recorded for flowers and fruits in some plants. Height (Ht_T1) was a measured variable consisting of continuous data, thus were analyzed using normal linear models (lm). The graphics were prepared using Grammar of Graphics (ggplot2) package [74].

## Supplementary Information

**Additional file 1.** 

**Additional file 2.** 

**Additional file 3.** 

**Additional file 4.** 

## Data Availability

All relevant datasets generated during and/or analysed during the current study can be found in online repositories and are available from Cassavabase, https://www.cassavabase.org/breeders/trial/7795?format = a website maintained by the Next Generation Cassava Breeding Project. The data may also be accessed at https://cassavabase.org/ftp/manuscripts/Baguma_et_al_2022. Additionally, the dataset files are included under “Supplementary files”.
